# Toward Smarter Orthopedic Care: Classifying Plantar Footprints from RGB Images Using Vision Transformers and CNNs

**DOI:** 10.3390/jimaging11110414

**Published:** 2025-11-16

**Authors:** Lidia Yolanda Ramírez-Rios, Jesús Everardo Olguín-Tiznado, Edgar Rene Ramos-Acosta, Everardo Inzunza-Gonzalez, Julio César Cano-Gutiérrez, Enrique Efrén García-Guerrero, Claudia Camargo-Wilson

**Affiliations:** Facultad de Ingeniería, Arquitectura y Diseño, Universidad Autónoma de Baja California, Ensenada 22860, Mexico; lidia.ramirez@uabc.edu.mx (L.Y.R.-R.); jeol79@uabc.edu.mx (J.E.O.-T.); edgar.rene.ramos.acosta@uabc.edu.mx (E.R.R.-A.); einzunza@uabc.edu.mx (E.I.-G.); jcano@uabc.edu.mx (J.C.C.-G.); eegarcia@uabc.edu.mx (E.E.G.-G.)

**Keywords:** artificial intelligence, RGB imaging, deep-learning, deep neural network, vision transformers, CNN, image-classification, medical imaging, footprint, hindfoot

## Abstract

The anatomical structure of the foot can be assessed by examining the plantar footprint for orthopedic intervention. In fact, there is a relationship between a specific type of foot and multiple musculoskeletal disorders, which are among the main ailments affecting the lower extremities, where its accurate classification is essential for early diagnosis. This work aims to develop a method for accurately classifying the plantar footprint and hindfoot, specifically concerning the sagittal plane. A custom image dataset was created, comprising 603 RGB plantar images that were modified and augmented. Six state-of-the-art models have been trained and evaluated: swin_tiny_patch4_window7_224, convnextv2_tiny, deit3_base_patch16_224, xception41, inception-v4, and efficientnet_b0. Among them, the swin_tiny_patch4_window7_224 model achieved 98.013% accuracy, demonstrating its potential as a reliable and low-cost tool for clinical screening and diagnosis of foot-related conditions.

## 1. Introduction

Foot diseases range in severity from benign problems, such as plantar warts and fungal infections, to severe disorders, including diabetic foot ulcers and peripheral artery disease. Proper foot care can help alleviate pain, prevent complications, enhance mobility, and improve overall quality of life. Therefore, the early and accurate diagnosis of foot disorders is essential, particularly for individuals with chronic conditions and musculoskeletal impairments.

Foot diseases may result from structural deformities or biomechanical issues, such as flat foot, cavus foot, valgus foot, or varus foot. These disorders can change foot mechanics, leading to pain and functional limitations [1]. Diagnosing these conditions accurately and promptly is crucial to stopping their progression and reducing the likelihood of complications [2].

Plantar footprint analysis helps healthcare professionals identify the foot type and its anatomical structure, essential for exploring and diagnosing lower extremity issues [3]. The plantar footprint reflects the pressure distribution and alignment of the foot, offering valuable diagnostic cues. Various plantar foot types exist, including planus, cavus, varus, valgus, and a regular foot. Each possesses distinct attributes, benefits, and drawbacks [4].

Pes planus, or flat feet, is a condition where the arch in the plantar region of the foot is diminished or absent, causing the sole to be in contact with the ground when standing upright [5]. The condition can be flexible [6,7] or rigid, depending on the degree of joint movement [8], and may be congenital or acquired due to ageing, injury, obesity, or disease [9]. Symptoms range from none to stiffness, fatigue, impaired balance, and an increased risk of injury.

Cavus foot, in contrast, is characterised by an abnormally high arch [10,11], which reduces ground contact. It may be hereditary or related to neurological or neuromuscular conditions, and patients often present with unstable gait, ankle sprains, and pain along the metatarsals or lateral border [12].

Varus foot causes an inward tilt of the heel and a raised medial arch [13]. It often leads to pressure overload on the medial side, resulting in discomfort, calluses, bunions, or hammertoes.

Conversely, a valgus foot is characterised by the heel tilting outward and the forefoot tilting inward. This results in a foot posture that is pronated, with a lower arch on the inside and a higher arch on the outside [14]. Genetics, neuromuscular disorders, skeletal abnormalities, or trauma can cause valgus deformity. This condition can lead to increased pressure on the outside of the foot, resulting in discomfort, calluses, corns, and blisters. Both valgus and varus deformities can also affect the alignment of the lower body, leading to complications in the knees, hips, and back. Therefore, identifying an individual’s foot type is essential for selecting suitable footwear, orthotic devices, exercise regimens, and therapeutic interventions to tackle foot-related issues [15,16].

Diagnostic imaging is crucial in accurately assessing the plantar foot type and advising on the appropriate measures to maintain optimal foot health [17]. However, traditional diagnostic techniques are often time-consuming, subjective, or limited in precision, motivating the search for automated and objective tools.

Recent advances in Artificial Intelligence (AI) and Deep Learning (DL) have revolutionized medical imaging by enabling automated, fast, and highly accurate image classification. Since the introduction of the perceptron in 1958 [18], AI has evolved significantly and is now widely applied in industrial automation [19,20,21,22,23,24], pharmaceuticals [25,26,27,28,29], and healthcare [30,31,32,33,34,35]. This evolution, as exposed in [36], reflects remarkable progress in algorithm design, computational resources, and data accessibility [37,38,39,40,41].

Precise evaluation of foot anatomy, especially in the plantar area, frequently depends on diagnostic imaging. Nonetheless, conventional foot analysis techniques possess constraints. Researchers have sought automated techniques to overcome these issues. Advancements in artificial intelligence, particularly in deep learning, have transformed image classification, facilitating faster and more precise identification of foot abnormalities.

Consequently, image classification for foot type has become an increasingly relevant research topic, given the enormous impact of foot disorders on a person’s quality of life. Utilising DL techniques for image analysis can aid in categorising foot ailments, facilitating the creation of diagnostic procedures that offer faster and more precise results [42].

### Motivation and Hypothesis

Traditional diagnostic methods for assessing foot types, such as X-ray imaging, plantar pressure sensors, or baropodometric platforms, are often prohibitively expensive, invasive, or inaccessible in specific clinical or remote environments. We propose that RGB images obtained from standard smartphones and podoscopes offer an economical, non-invasive option for automatic, real-time classification of foot shape through deep learning methodologies. This study argues that RGB-based datasets can serve as a dependable alternative, and the enhanced classification accuracy of our algorithms supports this assertion.

Given these limitations, this work aims to develop a robust methodology for the real-time classification of plantar footprints and hindfoot images using DL techniques. By leveraging RGB images, we propose a viable alternative for evaluating foot and ankle disorders without requiring radiographic imaging. This approach could empower healthcare professionals, especially in low-resource settings, to perform timely and accurate diagnostics.

The hypothesis underlying this research is that high-quality RGB images, when processed through advanced DL models, can accurately classify foot types, offering comparable or improved diagnostic performance over traditional imaging systems.

The structure of this paper is as follows: Section 2 reviews relevant literature on foot type classification. Section 3 presents the methodology, including dataset construction and the application of six state-of-the-art deep learning models through transfer learning. Section 4 details the performance outcomes, and Section 5 discusses their implications and potential applications. Finally, Section 6 summarises the key findings and outlines future directions.

## 2. Related Work

Several works have been conducted to analyse and classify images related to foot type [43,44,45,46,47]. A study presents the analysis of 103 radiographs of patients’ feet to classify them according to the height of the longitudinal arch defined by the angle of inclination of the calcaneus. Angular and linear measurements of the talus and calcaneus have been made on the radiographs. The measurements have been analysed using linear least squares regression to understand their relationship. As a result, not all measurements can predict arch height [48].

In a study utilising image processing methodologies, researchers examined the outcomes of 91 baropodometric examinations to create a labelling method employing the Python 3.10 programming language and functions from the Opencv library, including segmentation, geometric transformations, contour detection, and morphological procedures, such techniques were utilised to calculate the longitudinal arch index and to identify and classify foot types from the acquired pictures. The suggested approach for classifying foot deformities establishes a robust basis for subsequent research on enhancing classification precision [49].

In another study, a model was developed to classify foot types using numerical and foot pressure imaging data from 96 individuals. The authors fine-tuned VGG16, K-NN, and a stacking ensemble model. The ensemble model showed a better performance, 92% F1-score in diagnosing foot conditions [50].

Another study employed deep learning methods to classify foot types from plantar pressure images of 125 individuals, benchmarking ResNet-50 and YOLO-v5 with two configurations: the Squeeze-and-Excitation (SE) module and the Convolutional Block Attention module (CBAM). The results showed that ResNet outperformed YOLO-v5, achieving the highest accuracy of 82.6% [51].

Ref. [52] presents a deep learning-based approach for the automated diagnosis of uniquely flat feet from weight-bearing X-ray images and labelling by specialist radiologists, using the Support Vector Machine (SVM) as the primary classifier, with preprocessing using MobileNetV2 to extract deep features from images, achieving an accuracy of 95.1%.

In [53], the authors utilised 1-dimensional convolutional neural network with a sensor-enabled insole to distinguish standard, cavus, and planus feet. Such a sensor can measure angular velocity and force, which are combined to achieve better results with an accuracy of 100% in classifying among the three classes.

Some studies have also been performed to detect foot deformities such as hallux valgus. For this purpose, 507-foot images have been preprocessed using a convolutional neural network. The neural network obtained sufficiently accurate learning to differentiate images of feet with hallux valgus from those of standard feet [54].

While these studies demonstrate promising results using baropodometric, radiographic, pressure, or sensor-based images, they often require specialised equipment or clinical environments. This limits their scalability and accessibility in low-resource settings.

In contrast, the present work explores the feasibility of using RGB images—readily obtainable with smartphones and podoscopes—to classify foot types via deep learning models, offering a more affordable and non-invasive alternative.

As hypothesized in Motivation and Hypothesis, this paper aims to create, train, evaluate, validate, and test six classification models using deep learning techniques and propose a real-time plantar footprint classification system. This strategy can potentially increase diagnostic accuracy and efficiency, benefiting patient health. This research presents several innovative aspects in the domain of foot type classification. A custom dataset has been developed using a podoscope and a smartphone, comprising 603 images depicting diverse foot morphological characteristics. This novel strategy utilises readily available technologies to collect high-quality and varied data. The dataset has been enhanced using preprocessing and augmentation techniques, ensuring that the models were trained on a representative sample and improved their generalisation ability. A benchmark was conducted by training six state-of-the-art deep neural networks: swin_tiny_patch4_window7_224, convnextv2_tiny, deit3_base_patch16_224, xception41, inception-v4, and efficientNet_b0. Comprehensive optimisation of model performance has been achieved through extensive hyperparameter tuning, which involved adjusting the learning rate and batch size and implementing stratified k-fold cross-validation. In addition, the utilisation of Grad-CAM (Gradient-weighted Class Activation Mapping) for visualising the decision-making mechanism of the models provided an additional level of interpretability, providing valuable insights into the classification of various foot types by the models.

## 3. Materials and Methods

**Figure 1:**
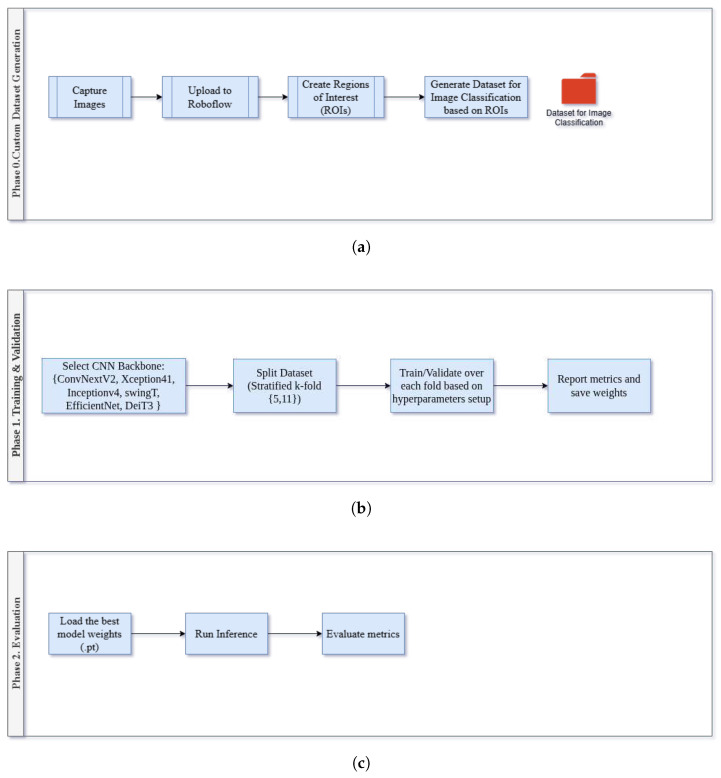
Overview of the deep learning workflow: (**a**) Phase 0—Custom dataset generation, (**b**) Phase 1—Training and validation process, and (**c**) Phase 2—Model evaluation.

**Figure 2:**
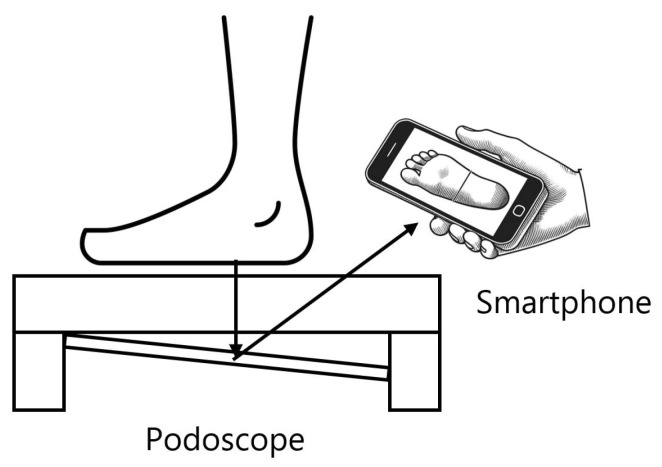
A patient over a podoscope and the smartphone are the main elements of the system.

**Figure 3:**
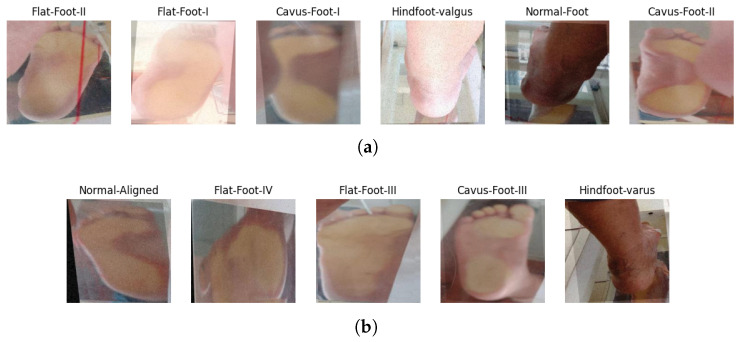
Samples from the original dataset prior to image data augmentation. (**a**) Top row shows Flat-Foot-II, Flat-Foot-I, Cavus-Foot-I, HindFoot-Valgus, Normal-Foot, and Cavus-Foot-II. (**b**) Bottom row shows Normal-Aligned, Flat-Foot-IV, Flat-Foot-III, Cavus-Foot-III, and Hindfoot-Varus.

The methodology has been divided into three phases. Phase 0, as depicted in Figure 1, involves creating the custom dataset. In contrast, Phase 1 selects the backbone Convolutional Neural Network (CNN) and applies the stratified k-fold cross-validation technique, as illustrated in Figure 2. The last phase, Phase 2, is all about model weights loading, running in inference mode, and metrics’ evaluation, as depicted in the following Figure 3:

The proposed methodology comprises three sequential phases, as outlined below:

- Phase 0—Custom Dataset Generation (Figure 1a):A custom dataset of RGB plantar footprint images was developed using a podoscope and a smartphone. The images were preprocessed and augmented to ensure sufficient variability and balance across foot types.
- Phase 1—Training and Validation (Figure 1b): Six state-of-the-art CNN architectures were selected as backbone models. Stratified *k*-fold cross-validation was employed to ensure robust evaluation across imbalanced classes.
- Phase 2—Evaluation (Figure 1c): Trained models were evaluated using accuracy, precision, recall, and F1-score. Models were also run in inference mode, and Grad-CAM visualisations were generated to interpret the classification results.

### 3.1. Phase 0. Custom Dataset Generation

For the initial stage, a podoscope has been utilised to stand a patient over the mirror as depicted in Figure 2. A smartphone was then used to capture plantar images, resulting in a dataset of 603 images, each with a resolution of 1200 × 1600 pixels. All these images have been uploaded to Roboflow [55] for manual annotation by defining the Region of Interest (ROI) for each image.

**Figure 4:**
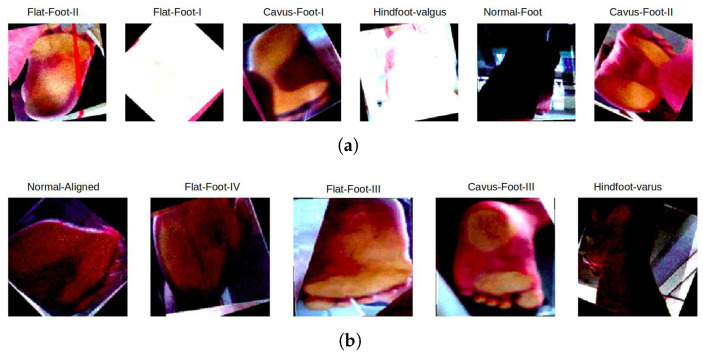
Samples from the dataset after image data augmentation. (**a**) Top row shows Flat-Foot-II, Flat-Foot-I, Cavus-Foot-I, HindFoot-Valgus, Normal-Foot, and Cavus-Foot-II. (**b**) Bottom row shows Normal-Aligned, Flat-Foot-IV, Flat-Foot-III, Cavus-Foot-III, and Hindfoot-Varus.

The original custom dataset has been labelled by three (3) experts in podology, who classified the images corresponding to eleven (11) classes as described in Table 1, while Figure 3 represents one sample per class:

To improve the generalisation capability of the models, the original dataset underwent preprocessing and data augmentation. Figure 4 shows samples after augmentation using the following transformations:

1. Resize: 224 px by 224 px.
2. Flip: Horizontal, Vertical.
3. 45° Random Rotation.
4. Saturation: Between −1% and +1%.
5. Brightness: Between −1% and +1%.
6. Contrast: Between −1% and +1%.

**Figure 5:**
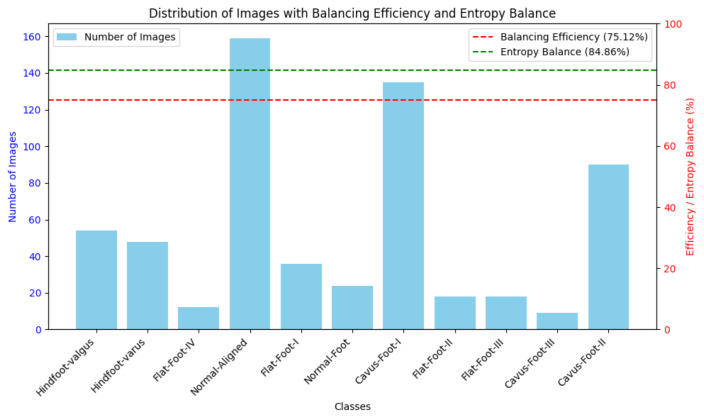
Balancing Efficiency (BE) and Entropy Balance (EB) from the dataset.

The distribution of the custom dataset is imbalanced as seen in Table 2 and Figure 5. Class No. 10 (Normal-Aligned) is the most represented with 159 samples, while Class No. 3 (Cavus-Foot-III) has only 9 samples. This yields an Imbalance Ratio (IR) of 17.67, calculated as

(1) $$\mathrm{IR}=\frac{I_{\max}}{I_{\min}}$$

Figure 5 illustrates the distribution and also reports the Balancing Efficiency (BE) and Entropy Balancing (EB), calculated using Equations (2) and (3), respectively. These metrics indicate the degree of balance within the dataset:

(2) $$\mathrm{BE}=\left(1-\frac{I_{\max}-I_{\min}}{\sum_{i=1}^{n}I_{i}}\right)\times 100$$

where $I_{\max}$ represents the maximum number of instances, while $I_{\min}$ corresponds to the minimum number of instances of the dataset.

(3) $$\mathrm{EB}=\frac{H}{H_{\max}}\times 100$$

where $H=-\sum_{i=1}^{n}p_{i}\log_{2}\left(p_{i}\right)$, $p_{i}=\frac{I_{i}}{\sum_{j=1}^{n}I_{j}}$, and $H_{\max}=\log_{2}\left(n\right)$.

**Figure 6:**
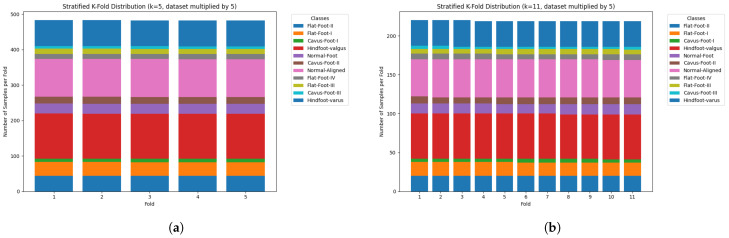
Training dataset distribution using stratified *k*-fold cross-validation and a 5× augmentation factor. (**a**) k=5 and (**b**) k=11.

Such metrics are summarised in the following Table 2:

To mitigate the class imbalance during model training, data augmentation was applied with multiplication factors of 5 and 10. Figure 6a,b show the dataset distribution using stratified *k*-fold cross-validation with k=5 and k=11, respectively, and a train/test split of 80/20, with only 80% under the k-fold cross-validation process and a 20% for the final test model evaluation.

**Figure 7:**
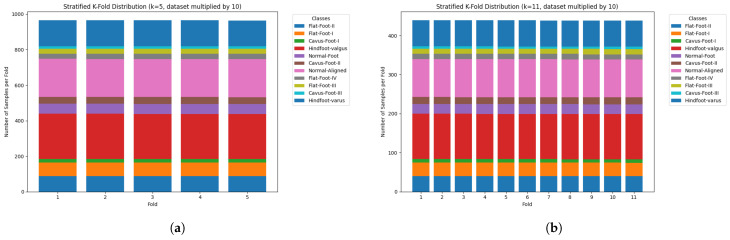
Training dataset distribution using stratified *k*-fold cross-validation and a 10× augmentation factor. (**a**) k=5 and (**b**) k=11.

Figure 7 shows an additional scenario with a 10× dataset multiplier. Figure 7a shows the distribution for k=5, and Figure 7b for k=11.

### 3.2. Phase 1. Training and Validation

The training code has been developed in Google Colaboratory, a Linux environment that hosts Python notebooks (Python 3.10). The training was performed on a high-performance A100 GPU (NVIDIA Corporation, Santa Clara, CA, USA) to get faster results. To optimise the performance of the classification models, a hyperparameter search was conducted. The parameters explored included batch size, number of folds for cross-validation (*k*), learning rate, and dataset multiplier (5× and 10×). The configuration used for fine-tuning is presented in Table 3.

Model performance was evaluated using five key metrics: Accuracy (Acc), Precision (*P*), Recall (*R*), F1-Score, and Matthews Correlation Coefficient (MCC). These metrics were chosen for their complementary nature, particularly in the context of imbalanced datasets. The total number of parameters of each model was also considered. Table 4 summarises the metrics and their respective definitions.

### 3.3. Pre-Trained Models

Six state-of-the-art (SOTA) architectures were assessed during the training phase to determine their performance. The architectures comprise: swin_tiny_patch4_window7_224 (denoted as M1), convnextv2_tiny (denoted as M2), deit3_base_patch16_224 (denoted as M3), xception41 (denoted as M4), inception-v4 (denoted as M5), and efficientNet_b0 (denoted as M6). The selected models, as recommended in [24], exemplify significant trends in deep computer vision over the past decade [56], highlighting advancements in CNNs and Vision Transformers (ViTs), which include enhancements in architecture and training methodologies.

#### 3.3.1. Inception-v4

Inception-v4 [57] is a purely Inception-based architecture without residual connections. It represents an evolution from Inception-v3, incorporating a larger number of inception modules and a more unified, linear structure. This model serves as the feature extractor of the network.

#### 3.3.2. Xception41

Xception [58] employs depthwise separable convolutions with residual connections. The architecture comprises 36 convolutional layers organised into 14 modules, most of which include linear residual links, except for the initial and final modules. Each separable convolution layer uses a depth multiplier of one, followed by batch normalisation.

#### 3.3.3. EfficientNet_b0

EfficientNet-B0 [59] is comparable to MnasNet [60] but slightly larger, targeting 400 million FLOPs (Floating-Point Operations per second). Its main component is the mobile inverted bottleneck (MBConv), which integrates squeeze-and-excitation mechanisms to weigh feature channels adaptively. This is analogous to Global Response Normalisation used in ConvNeXt, promoting contrast and selectivity.

#### 3.3.4. Deit3_base_patch16_224

The Data-efficient Image Transformer (DeiT) [61] improves ViT [62] by introducing a distillation token and teacher-student training. This setup allows transformers to learn efficiently from convolutional teachers on limited data.

DeiT3 [63] builds upon this by eliminating the distillation token and focusing on training optimisations, such as binary cross-entropy loss for ImageNet-1k, regularisation with LayerScale and stochastic depth, and the 3-Augment data augmentation scheme.

#### 3.3.5. Swin_tiny_patch4_window7_224

The Swin Transformer architecture [64] is a hierarchical vision transformer designed for a wide range of computer vision tasks, including classification, detection, and segmentation. Its two key innovations are the use of hierarchical feature maps and a shifted window approach for self-attention, which improves computational efficiency and locality.

#### 3.3.6. Convnextv2_tiny

ConvNeXt V2 [65] extends ConvNeXt [56] by integrating masked autoencoder (MAE) techniques for improved self-supervised learning. It features a Fully Convolutional MAE (FCMAE) framework using sparse convolutions to handle masked inputs efficiently and introduces Global Response Normalisation to counteract feature collapse.

All models were trained and validated using a stratified *k*-fold cross-validation approach, which ensures class balance in each fold and mitigates overfitting. Each sample appears in the training set at least once across *k* iterations, supporting robust generalisation. As illustrated in Figure 6 and Figure 7, this strategy preserved the original class distributions, enabling fair training even for under-represented classes [66,67,68,69,70].

This consistent validation across all architectures allowed a comprehensive and fair performance comparison.

### 3.4. Phase 2: Evaluation

During this phase, outlined in the subsequent Section 4, each model was assessed using the metrics and scores previously defined in Table 4. This section provides a thorough analysis of the experimental data, evaluating the efficacy of various deep learning architectures used for the classification task. The assessment conforms to established performance indicators, including Accuracy, Precision, Recall, F1-Score, and Matthews Correlation Coefficient (MCC), offering a comprehensive evaluation of overall predictive efficacy and class-specific performance. Models were trained with various hyperparameter configurations—modifying parameters such as learning rate, batch size, dataset multiplier, and training size—to determine optimal settings that improve the models’ generalisation to novel data.

The assessment incorporates both quantitative and visual methodologies to offer a comprehensive insight into model performance. Visualisations include parallel coordinates plots for hyperparameter tuning analysis, learning curves (for accuracy and loss) to evaluate convergence patterns, and validation metrics to measure generalisation gaps. Furthermore, model-agnostic insights were derived utilising ML-Explorer and Confusion-VIS, which statistically evaluate confusion matrices to assess similarity distances between models and emphasise per-class error distributions. The analysis includes Receiver Operating Characteristic (ROC) curves for each class to assess the trade-off between true positive and false positive rates, as well as comparisons of Top-1 Accuracy against model size to investigate the balance between model performance and computational complexity. Ultimately, interpretability was improved by (Grad-CAM), which visually emphasises the most significant regions in the input images, providing essential insights into whether the models concentrate on clinically pertinent areas during classification.

## 4. Results

This section presents the experimental results based on the evaluation metrics described previously. The training has been conducted under multiple hyperparameter configurations, while performance has been analysed using both visual and numerical approaches.

**Figure 8:**
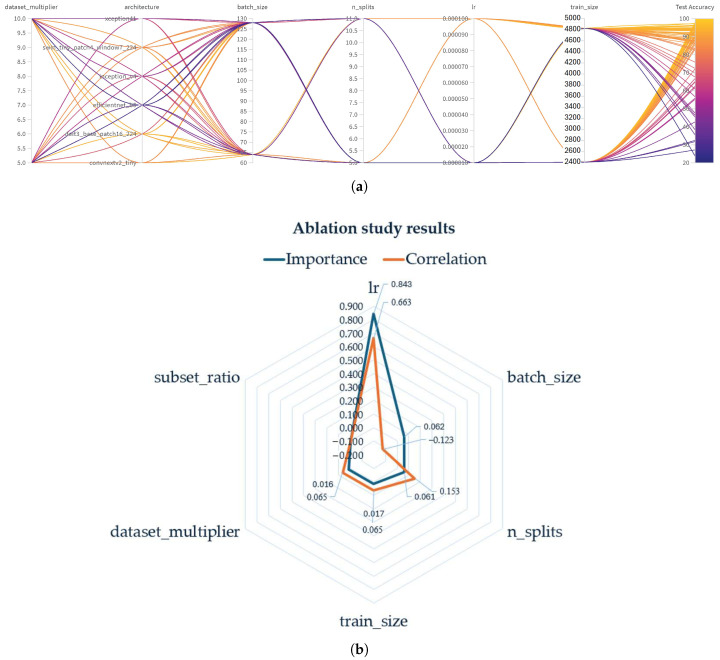
Training and validation metrics for all models: (**a**) Hyperparameter tuning results visualized through parallel coordinates, (**b**) Spider chart showing the importance and correlation of the ablation study results.

Figure 8a shows the outcome of hyperparameter tuning visualized through a parallel coordinates plot. The input dimensions include dataset_multiplier, architecture, batch_size, n_splits, learning rate (lr), and train_size. The Test_Accuracy is used as the target output, allowing comparison across trained models.

However, as shown in Table 5 and Figure 8b, the learning rate was the main parameter that optimized the process (importance = 0.843, correlation = 0.663), indicating its critical influence on test_accuracy. On the other hand, batch_size, the number of splits contributed less impact, while the dataset multiplier and subset ratio did not affect the test_accuracy.

**Figure 9:**
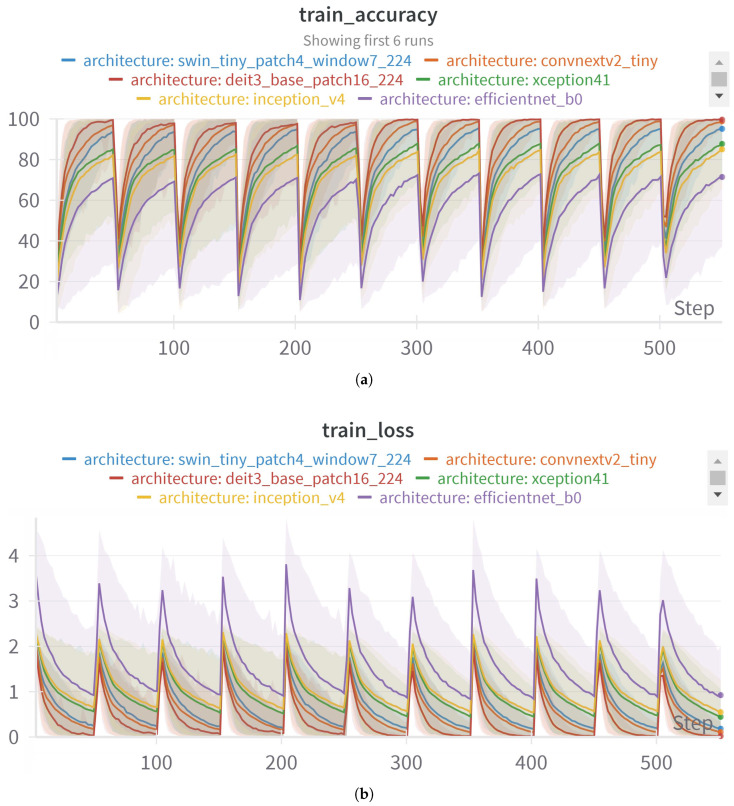

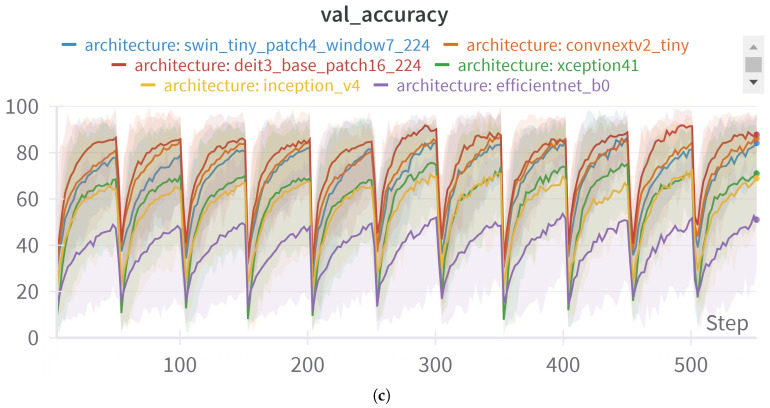
Training and validation metrics for all models: (**a**) train_accuracy refers to the accuracy during training phase, (**b**) train_loss depicts the loss during the training stage, and (**c**) val_accuracy shows the accuracy during validation.

Figure 9a exhibits a distinct rising trend in training accuracy for all assessed models, signifying successful convergence throughout the learning process. The swin_tiny_patch4_window7_224 and convnextv2_tiny architectures attain the maximum training accuracy, characterized by smoother and more stable learning curves.

Figure 9b demonstrates a steady reduction in training loss across the training iterations and k-folds, indicating peaks and valleys, but successful minimization of the objective function across all models. Nonetheless, models like efficientNet_b0 and xception41 exhibit erratic convergence patterns, possibly indicating heightened sensitivity to hyperparameter configurations or constrained representational capacity in the initial phases of training.

Figure 9c displays the validation accuracy, offering insight into the generalization efficacy of each architecture. The disparity between training and validation accuracy is particularly evident in specific models. The swin_tiny_patch4_window7_224 model consistently attains the most advantageous outcomes, exhibiting both elevated and stable validation accuracy. Conversely, models like xception41 and efficientNet_b0 demonstrate indications of overfitting, as seen by their robust performance on the training set alongside relatively diminished validation accuracy.

The final model’s performance has been evaluated using a test set of 302 images. Table 6 presents the best metrics obtained for each model, including Accuracy (Acc), Precision (P), Recall (R), F1-Score, Mathews Correlation Coefficient (MCC), and total parameters (Params). The top performer was swin_tiny_patch4_window7_224 (M1), with an accuracy of 98.01%, followed closely by convnextv2_tiny (M2) with 97.35% and deit3_base_patch16_224 (M3) with 97.18%. xception41 (M4) achieved 96.35%, while Inceptionv4 (M5) and efficientNet_b0 (M6), scored 93.53% and 91.37%, respectively.

**Figure 10:**
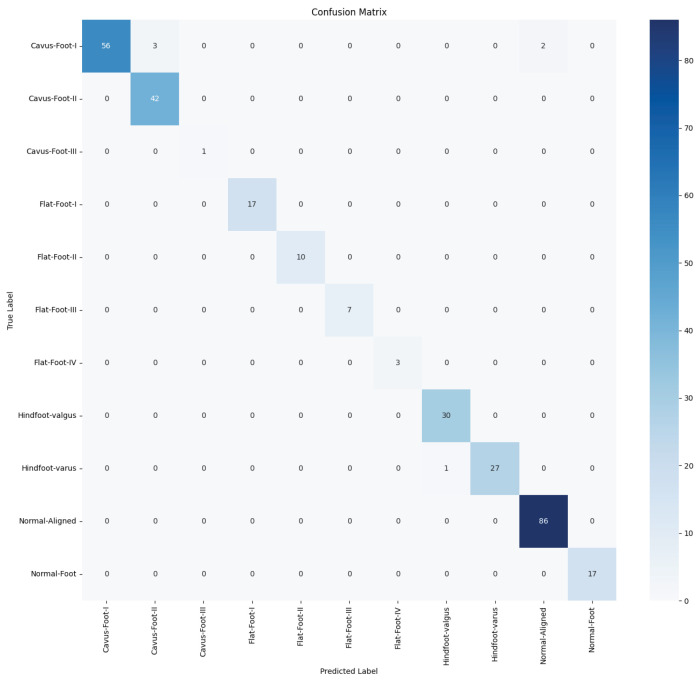
Confusion matrix for test set using swin_tiny_patch4_window7_224.

The confusion matrix of the best model, swin_tiny_patch4_window7_224, has been calculated over the test set, which is shown in Figure 10. The matrix presents the classification performance per class, with correct predictions along the diagonal.

**Figure 11:**
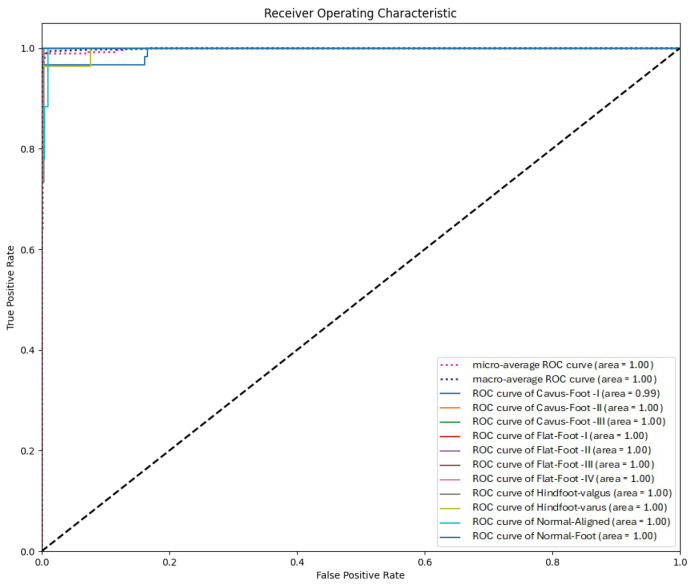
ROC curves by class using the swin_tiny_patch4_window7_224 model.

Figure 11 displays the Receiver Operating Characteristic (ROC) curve, showing the True Positive Rate (TPR) vs False Positive Rate (FPR) per class. While the model performs well for most classes, it exhibits difficulty accurately classifying samples from the class Cavus-Foot-I.

**Figure 12:**
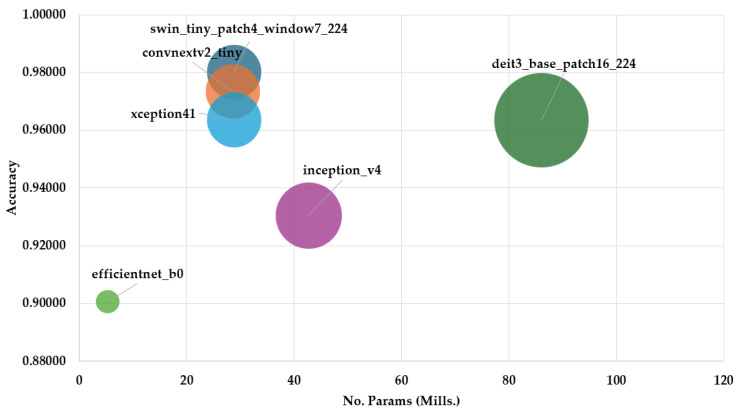
Top-1 Accuracy vs Model Size (parameters in millions).

Figure 12 compares the Top-1 Accuracy versus model size (in millions of parameters). The best model, swin_tiny_patch4_window7_224, achieved 98.01% accuracy with 28.8M parameters. convnextv2_tiny and deit3_base_patch16_224 also performed strongly, despite different model sizes. EfficientNet_b0 was the most lightweight model (5.3M) but with lower performance at 91.38%.

### 4.1. Confusion-VIS and ML-Explorer

Based on the methodology described in [71,72], a model-agnostic evaluation was conducted using statistical analysis of the confusion matrices for each model.

**Figure 13:**
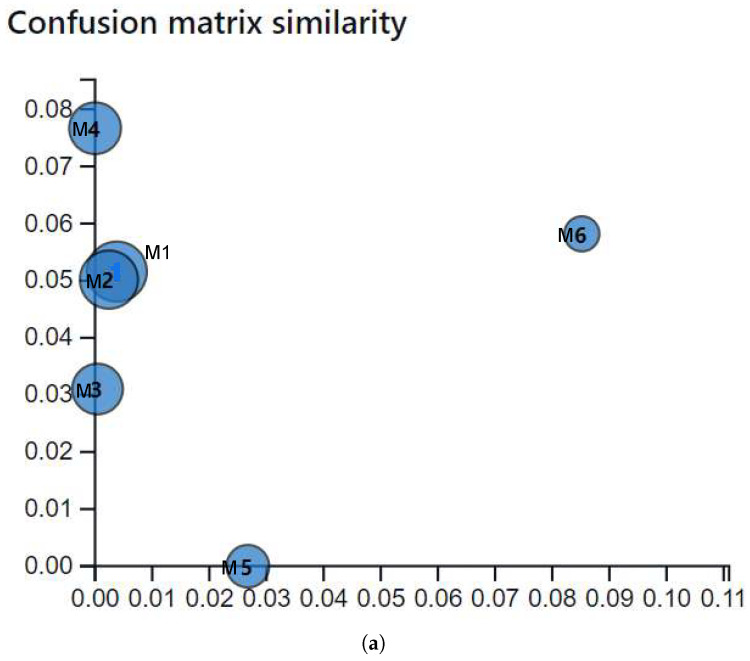

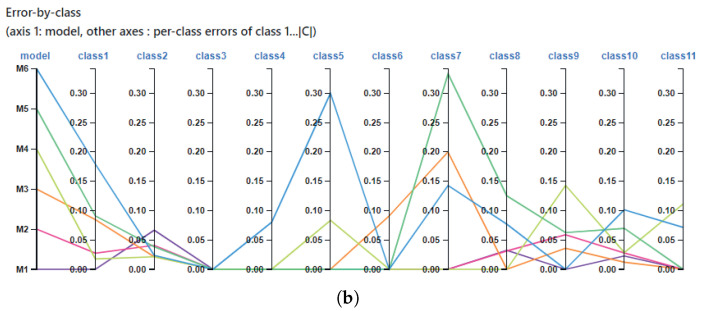
(**a**) Similarity distance among confusion matrices. (**b**) Error distribution per class for each model.

Figure 13a shows the similarity distances among confusion matrices. The models convnext v2_tiny and swin_tiny_patch4_window7_224 exhibit near-identical behaviour. Deit3_base _patch16_224, xception41, and inception_v4 show slightly higher divergence, with distances of 0.02, 0.03, and 0.055, respectively. In contrast, efficientNet_b0 stands out with the largest divergence (0.089) from the top models.

In Figure 13b, the model swin_tiny_patch4_window7_224 (M1) achieves a better classification with less than 0.06 error across three classes reporting minimal errors in class 2 (Cavus-Foot-II), class 8 (Hindfoot-valgus), and class 10 (Normal-Aligned), while efficientNet_b0 shows the greatest misclassifications across multiple classes, including classes 1, 2, 4, 5, 7, 8, 10, and 11.

**Figure 14:**
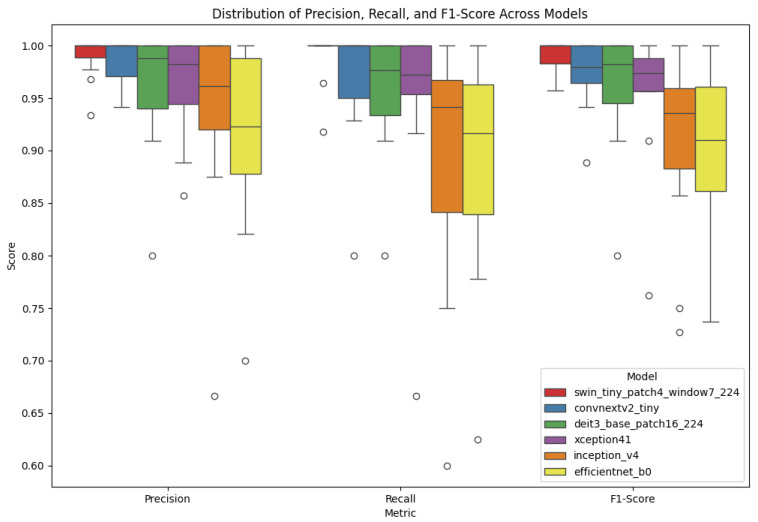
Boxplot of Precision, Recall, and F1-Score for all models.

Figure 14 shows a boxplot comparing Precision, Recall, and F1-Score across all six models. swin_tiny_patch4_window7_224 shows minimal variation, indicating stable performance. In contrast, efficientNet_b0, represented in yellow, exhibits high variance across metrics, highlighting its inconsistent behaviour.

### 4.2. Grad-CAM

**Figure 15:**
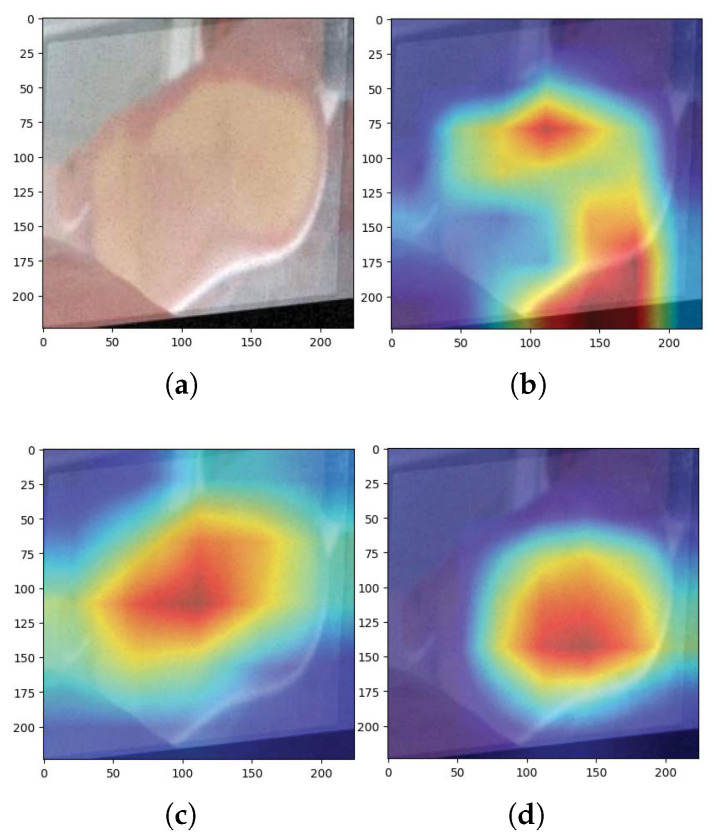
Grad-CAM results of different models on a test image: (**a**) original image, (**b**) efficientNet_b0, (**c**) inception-v4, and (**d**) xception41.

**Figure 16:**
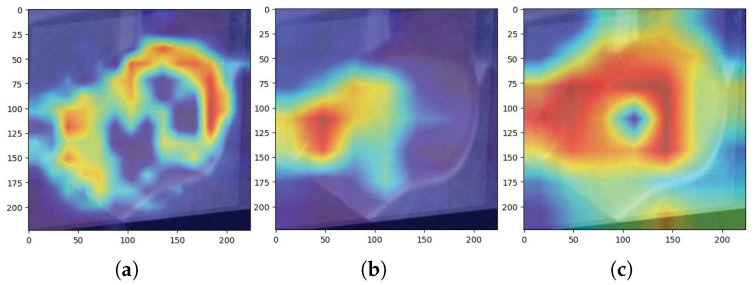
Grad-CAM results: (**a**) deit3_base_patch16_224, (**b**) convnextv2_tiny, (**c**) Swin_tiny_patch4_window7_224.

(Grad-CAM) is employed to interpret how trained models make classification decisions. It highlights the most influential regions of the input image by superimposing activation maps. Figure 15 and Figure 16 display examples of Grad-CAM visualisations for each model.

In Figure 15, the activation maps of efficientNet_b0, inception_v4, and xception41 are shown in comparison to the test input. Similarly, Figure 16 includes results from deit3_base_patch16_224, convnextv2_tiny, and swin_tiny_patch4_window7_224, showing how each model localises critical features for classification.

## 5. Discussions

One of the significant challenges in applying deep learning to foot disease classification is the lack of large, annotated image datasets. Building such datasets requires not only time and resources but also expert knowledge in podiatric conditions. Crowdsourcing platforms have emerged as a potential solution, enabling both patients and medical professionals to contribute images to centralised repositories. With access to diverse and well-labelled data, deep learning algorithms can be further improved, enhancing their diagnostic capabilities.

The error-by-class analysis revealed that models such as inception_v4 and efficientnet_b0 exhibited higher misclassification rates in specific classes. xception41 showed a notably high error in class 5 (Flat-Foot-II), which aligns with confusion matrix findings. Although efficientNet_b0 is computationally efficient, it presented higher overall error rates, reinforcing the trade-off between model complexity and performance. This highlights the necessity to balance lightweight architectures with classification accuracy, especially in scenarios where computational resources are constrained.

The boxplots comparing Precision, Recall, and F1-score demonstrate further discrepancies among models. Deit3_base_patch16_224 delivered consistently high performance, handling interclass variability effectively. In contrast, models like inception_v4 and efficientNet_b0 exhibited greater dispersion, indicating inconsistency in class-level predictions. These results emphasise the importance of multi-metric evaluation for selecting the optimal model, rather than relying solely on accuracy. The insights from the parallel coordinates plot and bubble chart reinforce this strategy, showing how architectural choices and hyperparameters influence both predictive performance and resource usage.

This study also compares favourably with prior works such as Chae [50] and Zhao [51], who utilised plantar pressure data and performed HSV transformations. In contrast, our approach relies solely on RGB images captured via smartphone and podoscope, simplifying acquisition and enhancing practicality in low-resource environments. Table 7 summarises the comparative metrics, using F1-Score as the principal criterion.

The key problem in this work was addressing the imbalance in the custom dataset, which exhibited a balancing efficiency of merely 75.12% (Figure 7). A Stratified K-Fold Cross-Validation technique was employed during training to maintain the original class distribution in each fold. This methodology allowed the models to proficiently learn from under-represented classes and resulted in consistently high performance across all criteria, notably with the swin_tiny_patch4_window7_224 model attaining 98.01% accuracy and an F1-Score of 97.99%.

This work is motivated by the constraints of conventional diagnostic techniques, such as X-ray imaging and plantar pressure platforms, which tend to be costly, invasive, and unavailable in resource-limited environments. Conversely, RGB imaging, obtained with a podoscope and smartphone, provides a non-invasive, economical option for assessing foot morphology. This corroborates our premise that RGB-based picture classification, combined with deep learning, can offer a dependable and scalable solution for foot type detection, facilitating greater clinical applications and real-time screening opportunities.

### 5.1. Scenario Usage

To overcome the existing limitations of deep learning-based foot disease classification, particularly the lack of extensive annotated datasets, a viable and scalable solution entails a hybrid imaging configuration that includes a podoscope for capturing the plantar surface and a webcam situated behind the patient to obtain sagittal and posterior views.

**Figure 17:**
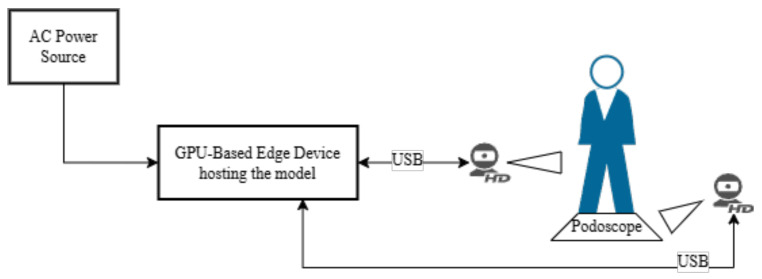
Block diagram of the proposed real-time system.

The block diagram shown in Figure 17 illustrates our proposal, which utilises a GPU-based edge device for real-time inference.

**Figure 18:**
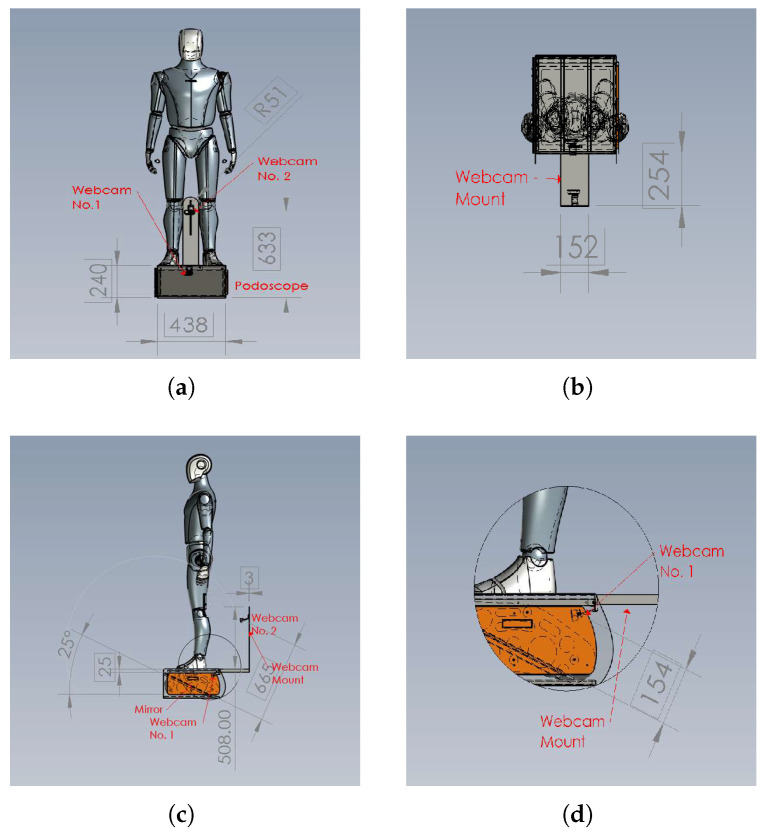
A patient stands on a podoscope to be diagnosed using two webcams. (**a**) Front View, (**b**) Right View, (**c**) Top View, and (**d**) Detail from Right View.

The patient stands barefoot on the podoscope platform, which utilizes an RGB camera positioned beneath a transparent surface to capture plantar images. As illustrated in Figure 18, all measurements are intended solely as references. Webcam 1 is positioned 154 mm from the mirror at a 25° angle relative to the ground. A second webcam is placed 633 mm above ground and 254 mm behind the patient to acquire sagittal and posterior perspectives of the heel and ankle. Both video streams are fed into a GPU-enabled PC that executes real-time inference, as detailed in Algorithm 1.
**Algorithm 1** Dual Webcam ROI Inference with GPU-Based Edge Device1:**Start**2:Initialize webcams3:Load trained model (best.pt)4:Start capture: Webcam 15:Start capture: Webcam 26:Define ROI from Webcam 17:Confirm with Enter8:Define ROI from Webcam 29:Confirm with Enter10:**while** System is Running **do**11:      Capture frame from Webcam 112:      Capture frame from Webcam 213:      Extract ROI from each frame14:      Preprocess each ROI15:      Send each ROI to GPU16:      Perform inference on ROI_1 and ROI_217:      Display results on screen18:**end while**19:Stop webcams20:**End**

### 5.2. Limitations and Future Work

Despite the suggested approach as a result for this work, the following limitations should be recognized:

- Limited population for dataset creation: The custom dataset was generated from limited cohort of participants, which restrict the diversity of plantar morphologies and gait patterns. Having a multi-site collaboration, can be a good solution to improve not only the number of participants, but age, races and diseases, increasing the model generalization.
- Restricted variation between classes: The differences between foot type classes, where unevenly distributed. Such disparity may result in the over-representation of specific of classes, while under-representation on others. Generative-AI (Gen-AI) can be a solution to generate synthetic images to increase the under-represented classes, or increase the size of the dataset.
- Extended dataset build process: The manual acquisition of images were labor-intensive hindering the research procedure. A full automated system for image acquisition and labeling can be a good approach to expedite this process.

On top of that, the suggested real-time dual-camera system exhibits significant potential for non-invasive foot type classification; however, multiple options for future investigation persist, such as the use of Image Segmentation technique, where details on the plantar foot can be exhibited.

For instance, integrating additional perspectives—such as oblique angles or dynamic gait video sequences—could expand the feature space and improve classification accuracy, particularly for complex deformities.

Another promising direction involves enriching the annotated dataset through Generative AI techniques to synthesize new samples for under-represented classes. This approach could significantly improve data diversity, enhance model generalisation, and reduce class imbalance.

Additionally, benchmarking performance across a range of GPU-enabled edge devices and comparing modern object detection frameworks would provide insights into the trade-offs between speed, accuracy, and hardware cost. Such efforts could also inform the design of mobile or wearable setups, reducing reliance on fixed camera configurations in clinical or remote environments.

Finally, the system’s real-world viability must be evaluated through clinical trials with podiatrists and longitudinal patient monitoring. These steps are critical to assess diagnostic effectiveness, ensure regulatory compliance, and support future deployment in healthcare settings.

## 6. Conclusions

The application of image classification for diagnosing foot-related conditions offers significant opportunities to enhance healthcare outcomes. By incorporating advanced image-based diagnostic tools, healthcare providers can improve the speed and accuracy of diagnoses. Early identification enabled by these methods facilitates timely intervention, minimising complications and improving patient prognoses. The adoption of such tools in clinical practice underscores their transformative potential in redefining traditional diagnostic workflows.

In this study, Deep Neural Networks (DNNs) played a central role in classifying foot types across a wide range of classes, surpassing previous works. The ability of DNNs to extract intricate visual patterns makes them particularly suitable for medical image analysis. Among the tested models, the swin_tiny_patch4_window7_224 architecture achieved the highest performance, reaching an accuracy of 98.013%. This result highlights the model’s capacity to reliably classify sagittal and plantar foot views, reinforcing the applicability of DNNs in foot diagnostics.

The fixed-camera setup with predefined Regions of Interest (ROIs) can be advantageous for foot type classification. This approach offers stability and simplifies training, which can be beneficial when deploying into real-time systems.

Nonetheless, a key limitation remains: the scarcity of large-scale, annotated foot image datasets. Constructing such datasets requires considerable time and domain expertise. Encouragingly, initiatives like crowdsourcing platforms are emerging to address this gap by enabling patients and clinicians to contribute labelled images to shared repositories. As these databases grow, so will the performance of deep learning models, enhancing diagnostic reliability.

In summary, this research demonstrates the feasibility and effectiveness of using DNNs with RGB-based imagery for automated, non-invasive foot classification. Continued efforts in dataset expansion, clinical validation, and system deployment will be essential to fully realise the benefits of AI-driven diagnostics in podiatric care.

## Figures and Tables

**Table 1:** Classes identified by foot type.

Class	Foot Type
Class 1	Cavus-Foot-I
Class 2	Cavus-Foot-II
Class 3	Cavus-Foot-III
Class 4	Flat-Foot-I
Class 5	Flat-Foot-II
Class 6	Flat-Foot-III
Class 7	Flat-Foot-IV
Class 8	Hindfoot-Valgus
Class 9	Hindfoot-Varus
Class 10	Normal-Aligned
Class 11	Normal-Foot

**Table 2:** Foot-type dataset insights.

Metric	Value
Max samples in a class ($I_{\max}$)	159 (Normal-Aligned)
Min samples in a class ($I_{\min}$)	9 (Cavus-Foot-III)
Imbalance Ratio (IR)	17.67
Balancing Efficiency (BE)	75.12%
Entropy Balancing (EB)	84.67%

**Table 3:** Parameters and hyperparameter values for the training process with 50 epochs.

Model	Learning Rate	Fold	Batch Size	Dataset Multiplier
[convnextv2_tiny, deit3_base_patch16, swin_tiny_patch4, xception41, inception_v4, efficientnet_b0]	[1 × 10^−5^, 1 × 10^−4^]	[5, 11]	[64, 128]	[5, 10]

**Table 4:** Comprehensive overview of classification metrics.

Metric	Description	Equation
Accuracy	The proportion of correct predictions (both true positives and true negatives) among the total number of cases. Most appropriate when the dataset is balanced.	$\frac{TP+TN}{TP+FP+TN+FN}$
Precision	Also known as the positive predictive value; measures the proportion of true positives among all positive predictions.	$\frac{TP}{TP+FP}$
Recall	Also known as sensitivity or true positive rate; measures the proportion of actual positives correctly identified.	$\frac{TP}{TP+FN}$
F1-Score	The harmonic mean of precision and recall; balances the trade-off between the two, especially useful in imbalanced datasets.	$\frac{2\cdot (Precision\cdot Recall)}{Precision+Recall}$
MCC	A balanced metric that accounts for all four confusion matrix categories; especially informative for imbalanced classes.	$\frac{TP\cdot TN-FP\cdot FN}{\sqrt{\left(TP+FP)(TP+FN)(TN+FP)(TN+FN\right)}}$

**Table 5:** An ablation study showing the relative importance and correlation of hyperparameters with respect to test_accuracy.

Parameter	Importance	Correlation
Learning rate (lr)	0.843	0.663
Batch size (batch_size)	0.062	−0.123
Number of splits (n_splits)	0.061	0.153
Training size (train_size)	0.017	0.065
Dataset multiplier (dataset_multiplier)	0.016	0.065
Subset ratio (subset_ratio)	0.000	0.000

**Table 6:** Best performance metrics for each model on the test set.

Model	Dataset Multiplier	n_Splits	Acc	P	R	F1-Score	MCC	Params (M)
M1	5	11	0.98013	0.98105	0.98013	0.97999	0.97639	28.8
M2	10	11	0.97347	0.97492	0.97347	0.97338	0.96838	28.6
M3	10	11	0.97181	0.97282	0.97181	0.97181	0.96662	86.1
M4	10	11	0.96352	0.96518	0.96352	0.96308	0.95630	28.8
M5	10	5	0.93532	0.93631	0.93532	0.93538	0.92370	42.7
M6	10	11	0.91376	0.91494	0.91376	0.91356	0.89483	5.3

**Table 7:** Results compared to other related works.

Research Work	Classes	Image Source	Original Dataset	Augmented Dataset	F1-Score
Chae [50]	3	Pressure Sensors (HSV)	192	36.8 K	0.9255
Zhao [51]	3	Plantar Pressure Plate System	1573	Not Published	0.9230
This work	11	Podoscope and camera (RGB)	603	3 K	0.9799

## Data Availability

The data presented in this study are available by reasonable request from the corresponding author.
